# A Poly-Glutamine Region in the *Drosophila* VAChT Dictates Fill-Level of Cholinergic Synaptic Vesicles

**DOI:** 10.1523/ENEURO.0477-18.2019

**Published:** 2019-03-04

**Authors:** Samuel W. Vernon, Jim Goodchild, Richard A. Baines

**Affiliations:** 1 Division of Neuroscience and Experimental Psychology, School of Biological Sciences, Faculty of Biology, Medicine and Health, University of Manchester, Manchester Academic Health Science Centre, Manchester M13 9PL, United Kingdom; 2 Syngenta Ltd, Bracknell, Berkshire RG42 6EY, United Kingdom

**Keywords:** acetylcholine, *Drosophila*, neurotransmitter, synapse, synaptic vesicle, transporter

## Abstract

While the primary role of vesicular transporters is to load neurotransmitters into synaptic vesicles (SVs), accumulating evidence suggests that these proteins also contribute to additional aspects of synaptic function, including vesicle release. In this study, we extend the role of the VAChT to include regulating the transmitter content of SVs. We report that manipulation of a C-terminal poly-glutamine (polyQ) region in the *Drosophila* VAChT is sufficient to influence transmitter content, and release frequency, of cholinergic vesicles from the terminals of premotor interneurons. Specifically, we find that reduction of the polyQ region, by one glutamine residue (*13Q* to *12Q*), results in a significant increase in both amplitude and frequency of spontaneous cholinergic miniature EPSCs (mEPSCs) recorded in the aCC and RP2 motoneurons. Moreover, this truncation also results in evoked synaptic currents that show increased duration: consistent with increased ACh release. By contrast, extension of the polyQ region by one glutamine (*13Q* to *14Q*) is sufficient to reduce mEPSC amplitude and frequency and, moreover, prevents evoked SV release. Finally, a complete deletion of the polyQ region (13Q to 0Q) has no obvious effects to mEPSCs, but again evoked synaptic currents show increased duration. The mechanisms that ensure SVs are filled to physiologically-appropriate levels remain unknown. Our study identifies the polyQ region of the insect VAChT to be required for correct vesicle transmitter loading and, thus, provides opportunity to increase understanding of this critical aspect of neurotransmission.

## Significance Statement

Neurotransmitter loading of synaptic vesicles (SVs) is tightly regulated and underpins the quantal theory of neurotransmission. However, although observed at every synapse studied, the mechanistic basis that ensures vesicle-filling stops at a fixed, pre-determined, level remains poorly understood. In this study we identify a C-terminal poly-glutamine (polyQ) region in the *Drosophila* VAChT to be critical for vesicle loading of ACh. Reduction or extension of this region, by just one glutamine residue, is sufficient to increase or decrease, respectively, the amount of ACh loaded. Our work significantly advances the field of synaptic physiology by identifying a region of a vesicular transporter that regulates the extent to which SVs are filled.

## Introduction

Vesicular loading of synaptic vesicles (SVs) is dependent on initial acidification mediated by the vATPase pump. This pump generates both a pH gradient (ΔpH) and a voltage gradient (Δψ) across the SV membrane (Edwards, 2007; Takamori, 2016). The relative requirement for these two components for loading is dependent on neurotransmitter: anionic transmitters such as glutamate rely more heavily on Δψ (Maycox et al., 1988; Schenck et al., 2009; Takamori, 2016). Zwitterionic transmitters require both gradients (Edwards, 2007; Takamori, 2016), whereas, cationic transmitters (e.g., ACh) rely predominantly on ΔpH (Parsons et al., 1993; Parsons, 2000; Takamori, 2016). Transport of ACh into a SV involves the exchange of two protons in an antiporter system using the proton-electrochemical gradient (Südhof, 2004; Lawal and Krantz, 2013). The current model suggests that one proton is used to transport ACh into the SV lumen while the second proton is needed to re-orientate the VAChT substrate binding site back toward the cytoplasm (2H^+^ for 1ACh^+^; Parsons, 2000). *In situ*, acidified SVs exhibit a pH ∼1.4 units less than un-acidified SVs (Parsons, 2000). Theoretically, the cholinergic SV lumen has the capacity to concentrate ACh by 100-fold relative to cytoplasmic levels (which range between 1 and 4 mM; Parsons et al., 1993; Parsons, 2000). However, the maximal reported accumulation of ACh in SVs has been found to saturate at ∼4 mM, suggesting a rather dramatic (and unknown) limiting factor impedes loading (Parsons et al., 1993; Varoqui and Erickson, 1996; Parsons, 2000).

A key limiting factor may be copy number of functional transporter per SV. Murine and *Drosophila* NMJ and mammalian cell culture models suggests vesicular loading is altered following either genetic and/or pharmacological manipulation of transporter activity (Varoqui and Erickson, 1996; Song et al., 1997; Daniels et al., 2004; Wilson et al., 2005; Prado et al., 2006; De Castro et al., 2009; Lima et al., 2010). However, it is notable that upregulation of VAChT expression fails to show effects to quantal size at either snake NMJ or *Drosophila* motoneurons that receive cholinergic excitation (Parsons et al., 1999; Cash et al., 2016). An inability of increased transporter to affect SV loading is consistent with a set-point model of filling (Williams, 1997; Cash et al., 2016). This model posits that SVs fill to a predetermined level, independent of filling rate, which changes following manipulation of transporter expression level. We have previously reported that transgenic expression of VAChT, which carries a single glutamine truncation in a C-terminal poly-glutamine (polyQ) region (13Q to 12Q), results in increased quanta of spontaneously released SVs at identified interneuron to motoneuron synapses (Cash et al., 2016). This region, therefore, may contribute to the mechanism that regulates SV loading.

Here, we use electrophysiological characterization of cholinergic release at *Drosophila* larval and embryonic interneuron→motoneuron synapses to investigate the physiologic implications to SV loading when the VAChT C-terminal polyQ region is manipulated. We find, in agreement with previously published literature, that expression of a single glutamine truncation VAChT^12Q^ increases both amplitude and frequency of spontaneously released cholinergic miniature EPSCs (mEPSCs; i.e., individual SV release) recorded from aCC and RP2 motoneurons. Evoked synaptic currents also show an increased duration consistent with an increased ACh load. Conversely, we further show that CRISPR induced single amino acid extension of the polyQ region (VAChT^14Q^) results in the opposite effect: reduced mEPSC amplitude and frequency and, moreover, an inability to support evoked release. CRISPR mediated deletion of the polyQ region (VAChT^ΔQ^) has no effect on mEPSC kinetics suggesting that elongation or truncation of the VAChT polyQ region is more detrimental to cholinergic functioning than its removal.

## Materials and Methods

### Fly stocks

Flies were maintained under standard conditions at 25°C. GAL4 drivers used to recapitulate expression of the cholinergic locus were *cha^B19^*(Salvaterra and Kitamoto, 2001) and *ChAT-BAC* (gifted by Steve Stowers, Montana State University). These lines were used to drive expression of *UAS-VAChT^12Q^*(Cash et al., 2016), UAS-*ChR2^ChETA^* (Bloomington 36354; Gunaydin et al., 2010), and UAS-*ChR2* (Pulver et al., 2009). CRISPR constructs were prepared as described below and injected into c*as9*-expressing embryos (*yw*; attP40 nos-cas*9*/*CyO*;*+*) by BestGene Inc. Control lines were the cleaned CRISPR-injected line lacking construct insertion (*w^-^*; +; +). Animals used were of either sex.

### gRNA and insert design, template oligo and plasmid construction

The CRISPR Optimal Target Finder tool (http://tools.flycrispr.molbio.wisc.edu/targetFinder/) was used to specify target cut sequence specificity (GATTACCGCTATCAGGTACC). Two guide RNA constructs were made to generate cuts in 5’- and 3’-UTR of *VAChT*, respectively. The gRNA oligonucleotides (5’ to 3’) are: 5’-UTR: CTTCGAGAGGAAGTCCCAAAGAAAC and AAACGTTTCTTTGGGACTTCCTCTC; 3’-UTR: CTTCGTATTATTACTATAGACATAT and AAACATATGTCTATAGTAATAATAC, sense and antisense, respectively). A total of 100 pmol of each 5’ phosphorylated sense and antisense gRNA oligonucleotides were mixed, denatured at 95°C and then reduced to 25°C at a rate of –0.1°C/s and ligated to the guide RNA expression plasmid, pU6-BbsI-chiRNA (plasmid #45946, Addgene). Oligos used to generate PAM and polyQ site mutations are shown in Table 1. Briefly, for 5’ PAM site mutagenesis, PCR of primers a + b and c + d (containing TGG to TGC point mutations) were run against *Drosophila* genomic DNA (PAM) or *VAChT* plasmid DNA (polyQ; PCR1). Following purification, PCR products (a + b and c + d) were used as templates for a second PCR using the most 5’ and 3’ primers of PCR1 (primers a and d, Table 1). This process was repeated for 3’ PAM site mutagenesis utilizing primers (e + f and g + h), *VAChT^ΔQ^* (i + j and k + l) and *VAChT^14Q^*(m + n and o + p). Full UTR sequence with PAM mutations were purified, sequenced and mobilized to pHD-DsRed (plasmid #51434, Addgene) as a dsDNA donor template for CRISPR/Cas9-mediated homology-directed repair (HDR) using restriction digests (5’ = AscI and BssSI; 3’ = SpeI and XhoI). PolyQ products were first mobilized to the cloning vector pJET1.2, then to *VAChT* containing pBSII (BamH1 and NDe1; Cash et al., 2016) then finally to pHD-DsRed with (EcoR1 and NDe1). Sequence was checked by Sanger sequencing at the Manchester Sequencing Facility. Positive progeny was identified by the expression of DsRed in larvae following the 3xP3 expression pattern. Lines were cleaned and balanced by BestGene. Sequences were re-confirmed at the Manchester Sequencing facility before experimentation.

**Table 1.** Primers used for creation of *Drosophila VAChT* UTR with modified PAM sites (5’: a, b, c, d and 3’: e, f, g, h) and modified PolyQ regions (5’: i, j, k, l and 3’: m, n, o, p)

Sequence	Use		
ATCGGGCGCGCCGAATTCATGCTTGGGTCGACTTAAGCTC	a	a + b	(5’PAM)
ACAAAGTTCTGATGCAGTTTCTTTGG	b		
CCAAAGAAACTGCATCAGAACTTTGT	c	c + d	
CTTAAATAGTCGGGTATAATCGGTACTA	d
GTACACTAGTTCGTGTTCTTTTGCACACCTCC	e	e + f	(3’PAM)
ACGTACCACTTGGCTATATGTCTATA	f		
TATAGACATATAGCCAAGTGGTACGT	g	g + h	
GCTACTCGAGAAGTCCGCCACAATGACAACC	h
GTGCCTACTGGACGGGCT	i	i + j	*VAChT^ΔQ^*
CAGGACCTCTGCTCTGGACGAAGGGATTGGCCACACGG	j		
CCGTGTGGCCAATCCCTTCGTCCAGAGCAGAGGTCCTG	k	k + l	
GCTATTAATTAACATATGTAGGAGTATCTGTTCGGGGCAA	l
GTGCCTACTGGACGGGCT	m	m + n	*VAChT^+Q^*
CTGCTGCTGCTGCTGTTGTTGTTGCTGCTGCTGCTGCTGCTG	n		
CAGCAGCAGCAGCAACAACAACAACAACAGCAGCAGGTCCAGAGC	o	o + p	
GCTATTAATTAACATATGTAGGAGTATCTGTTCGGGGCAA	p

### Quantitative RT-PCR (qRT-PCR)

A total of 50 late stage 17 embryos (per replicate) were collected. RNA was extracted using the RNeasy micro kit (QIAGEN). Single strand cDNA was synthesized using the Revert Aid H minus first strand cDNA synthesis kit (Fermentas). qRT-PCR was performed using a LightCycler480 II (Roche) with SYBR Green I Master reaction mix (Roche). The thermal profile used was 10 s at 72°C. Single-product amplification was completed by post-reaction dissociation analysis. PCR primers were designed with the aid of LightCycler Probe Design Software 2.0 (v1.0; Roche). Results were analyzed by the 2^-ΔΔCt^ method. Ct values used were means of two to three independent replicates. Gene expression was normalised to actin. Primers (5’ to 3’) were as follows: actin, CTTCTACAATGAGCTGCGT and GAGAGCACAGCCTGGAT; VAChT,CTCATCCTCGTGATTGTA, and ACGGGTATGATCTTTCC.

### Larval and embryonic whole-cell patch-clamp recordings

Recordings were performed at room temperature (20–22°C). Third-instar larvae were dissected in external saline (135 mM NaCl, 5 mM KCl, 4 mM MgCl_2_·_6_H_2_O, 2 mM CaCl_2_·2H_2_O, 5 mM N-Tris[hydroxymethyl]methyl-2-aminoethanesulfonic acid, and 36 mM sucrose; pH 7.15). The CNS was removed and secured to a Sylgard (Dow-Corning)-coated coverslip using tissue glue (GLUture; WPI). The neurolemma surrounding the CNS was partially removed using protease (1% type XIV; Sigma) contained in a wide-bore (15 μm) patch pipette. Whole cell recordings were conducted using borosilicate glass electrodes (GC100TF-10; Harvard Apparatus), fire-polished to resistances of between 7 and 10 MΩ for L3 recordings and 14–18 MΩ for embryonic recordings. The aCC/RP2 motoneurons were identified by characteristic soma size and position within the ventral nerve cord. Cell identity was sporadically confirmed, after recording, by filling with 0.1% Alexa Fluor 488 hydrazyde sodium salt (Invitrogen), included in the internal patch saline (140 mM potassium gluconate, 2 mM MgCl_2_·_6_H_2_O, 2 mM EGTA, 5 mM KCl, and 20 mM HEPES; pH 7.4). Tetrodotoxin (TTX; 2 μM, Alomone Labs) was included in the external saline to block action potential-induced SV release. Recordings were made using a MultiClamp 700B amplifier. Cells were held at –60 mV and recordings were sampled at 100 kHz and lowpass filtered at 0.5 kHz, using pClamp 10.6 (Molecular Devices). Only neurons with an input resistance of ≥500 MΩ (L3 recordings) or ≥1 GΩ (embryo) were accepted for analysis. Evoked vesicle exocytosis was elicited through driving UAS-ChR2 or UAS-ChR2^ChETA^ using blue light (λ470 nm, 10 ms, 1 Hz/0.05 Hz, light intensity 9.65 mW/cm^2^).

### Statistics

Statistical significance between group means was assessed using either a Student’s *t* test (where a single experimental group is compared to a single control group), a one-way ANOVA followed by Bonferroni’s *post hoc* test (multiple experimental groups). In all tests, confidence intervals of *p ≤ 0.05, **p ≤ 0.01, ***p ≤ 0.001, and ****p ≤ 0.0001 were used for significance. Data shown are mean ± SEM.

## Results

### VAChT^12Q^ increases SV loading at cholinergic synapses

**Figure 1.**
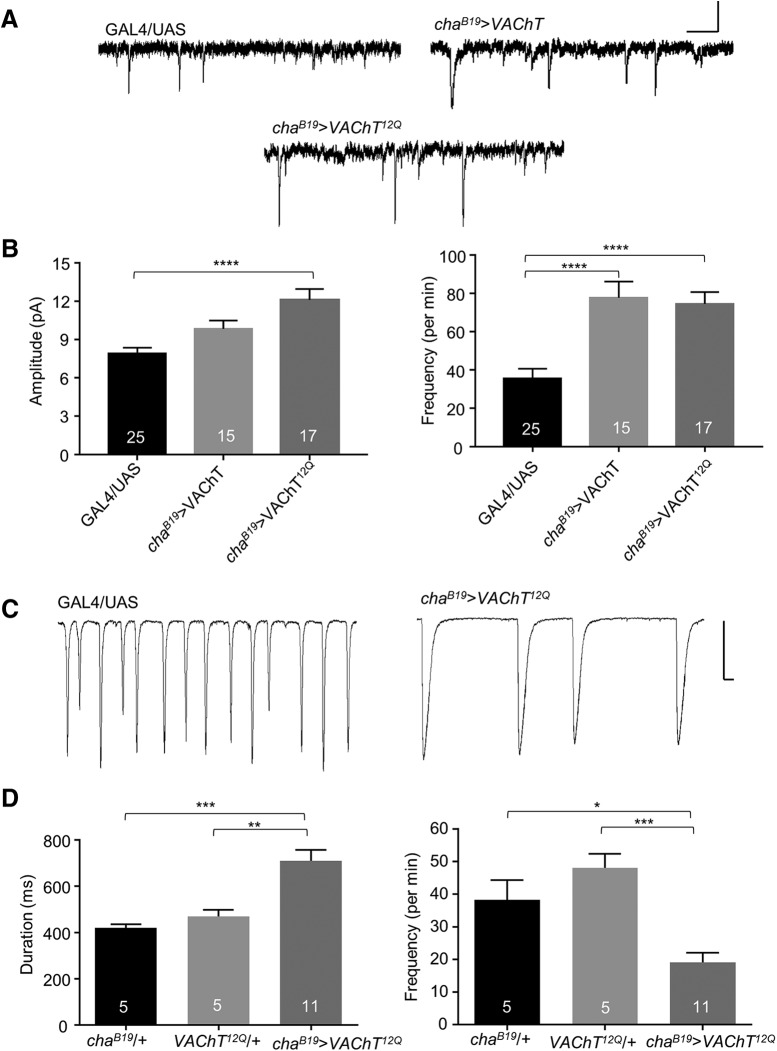
Expression of *VAChT^12Q^* increases mEPSC amplitude. ***A***, Representative traces of mEPSCs recorded from L3 aCC/RP2 in GAL4 (shown) and UAS (not shown) controls and following expression of *VAChT^12Q^* in all cholinergic neurons (*cha^B19^*>VAChT^12Q^). Scale bar: 10 pA/30 ms. ***B***, *VAChT* ^12Q^ increases both mEPSC amplitude: *p* < 0.0001 and frequency: *p* = 0.0001. Whereas expression of wild-type *VAChT* increases mEPSC frequency *p* < 0.0001 but not amplitude (*p* = 0.1). ***C***, Representative SRCs recorded from L3 aCC/RP2 in GAL4 (not shown) and UAS (shown) controls and *cha^B19^*>VAChT^12Q^. Scale bar: 400 pA/500 ms. ***D***, Following expression of VAChT^12Q^, SRCs show significantly increased duration (*cha^B19^*/+: *p* = 0.001, UAS/+: *p* = 0.005) and reduced frequency (*cha^B19^*/+: *p* = 0.01, UAS/+: ***p = 0.0003) with no effect to amplitude (*p* = 0.23). All data points are mean ± SEM, *n* is stated in each bar. *p ≤ 0.05, **p ≤ 0.01, ****p ≤ 0.0001.

We undertook patch-clamp recordings from well-characterized aCC/RP2 motoneurons, which receive identical cholinergic synaptic input (Baines et al., 1999). We recorded spontaneous mEPSCs, achieved by blocking action potential-dependent activity with TTX. We have previously shown that expression of transgenic VAChT^12Q^, in a wild-type background (i.e., VAChT^13Q^), significantly increases mEPSC amplitude and release frequency (Cash et al., 2016). It should be noted that, unlike the NMJ, mEPSCs recorded in central neurons can (and in this case do) show a range of amplitudes due to filtering of current spread through axonal and dendritic regions. In this study, we confirm that transgenic expression of VAChT^12Q^ increases mEPSC amplitude (7.9 ± 0.5 vs 12.1 ± 0.8 pA, GAL4/UAS vs *cha^B19^*>VAChT^12Q^, respectively, *p* < 1 × 10^−4^; Fig. 1*A*,*B*) and also frequency (35.5 ± 5.1 vs 74.3 ± 6.2 per min, GAL4/UAS vs *cha^B19^*>VAChT^12Q^, respectively, *p* = 1 × 10^−4^). By contrast, upregulation of wild-type VAChT did not significantly increase mEPSC amplitude (7.9 ± 0.5 vs 9.9 ± 0.6 pA, GAL4/UAS vs *cha^B19^*>VAChT, respectively, *p* = 0.10; Fig. 1*A*,*B*). However, in line with VAChT^12Q^ upregulation, frequency was increased (35.5 ± 5.1 vs 77.7 ± 8.5 per min, GAL4/UAS vs *cha^B19^*>VAChT, respectively, *p* < 1 × 10^−4^). These data suggest that manipulation of the polyQ region, rather than expressional regulation of VAChT, regulates cholinergic SV loading.

To determine whether the effects we observed in mEPSCs, following expression of VAChT^12Q^, affect evoked release we recorded evoked spontaneous rhythmic currents (SRCs) in aCC/RP2 (i.e., in the absence of TTX). Figure 1*C*,*D* shows that SRCs are supported, but that they exhibit altered kinetics: specifically showing significantly increased duration [420.3 ± 14.5, 468.8 ± 27.9 vs 709.2 ± 47.4 ms, *cha^B19^/+*, UAS/+ vs *cha^B19^*>*VAChT^12Q^*, respectively, *p* = 1 × 10^−3^ (*cha^B19^/+*) and 5 × 10^−3^ (UAS/+)]. SRC frequency was also significantly reduced [38.3 ± 5.9, 48.1 ± 4.2 vs 19.0 ± 3.0 per min, *cha^B19^/+*, UAS/+ vs *cha^B19^*>*VAChT^12Q^*, respectively, *p* = 1 × 10^−2^ (*cha^B19^/+*) and 3 × 10^−4^ (UAS/+)], while amplitude remained unchanged (*p* = 0.23).

**Figure 2.**
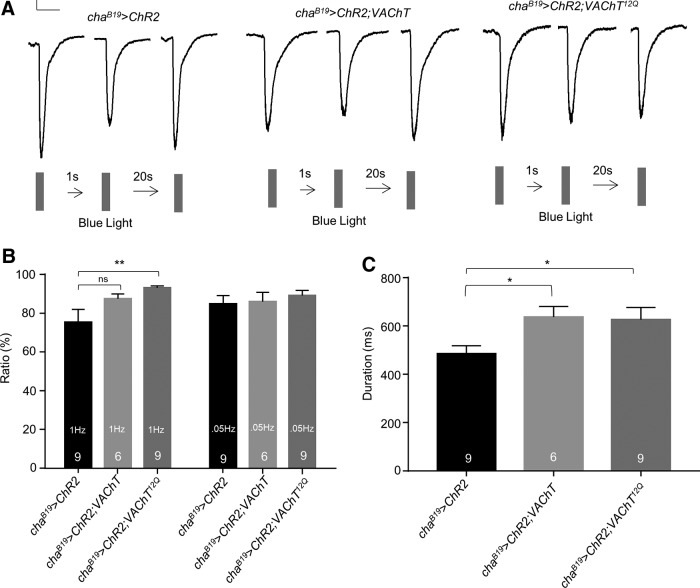
Expression of *VAChT^12Q^* increases optogentically-evoked EPSC duration. ***A***, Traces of EPSCs recorded from L3 aCC/RP2 in control (*cha^B19^>ChR2*) versus experimental (*cha^B19^>ChR2; VAChT* or *cha^B19^>ChR2; VAChT^12Q^*) conditions. EPSCs shown are composite averages derived from 9, 6 and 9 cells respectively. Scale bar: 50 pA/500 ms. ***B***, Paired-pulse stimulations, at 1 Hz, show the presence of *VAChT^12Q^* enables presynaptic release to resist run-down that occurs in the control (*p* = 0.007). This is not seen in wild-type *VAChT* expression (*p* = 0.13). This effect is abrogated when the second stimulus is applied at 0.05 Hz (*p* = 0.92). ***C***, Expression of *VAChT^12Q^* increased duration of optogenetically-evoked EPSCs (*p* = 0.03) but did not influence amplitude (*p* = 0.81). Expression of wild-type *VAChT* also increased EPSC duration (*p* = 0.02), again with no effect on amplitude (*p* = 0.49). All data points are mean ± SEM, *n* is stated in each bar. *p ≤ 0.05, **p ≤ 0.01.

As can be seen in Figure 1*C*, network-driven SRCs show variability in amplitude, perhaps due to differential activity of premotor interneurons and/or filtering of current spread through the dendritic regions of motoneurons. To provide a more rigorous baseline (i.e., to reduce variability particularly in amplitude) we used an optogenetic approach. This is sufficient to produce EPSCs that are more consistent in amplitude, and are identical to SRCs (but as these are not spontaneous, we term them EPSCs). We expressed ChR2 (Pulver et al., 2009) in all cholinergic neurons using Cha^B19^ GAL4 (this includes the excitatory premotor interneurons to aCC/RP2). Expression of *VAChT^12Q^* similarly increased duration of optogenetically-evoked EPSCs (485.4 ± 32.9 vs 625.9 ± 49.9 ms, *cha^B19^>ChR2* vs *cha^B19^>ChR2; VAChT^12Q^*, respectively, *p* = 0.03) but again did not influence amplitude (23.4 ± 2.7 vs 24.5 ± 3.3 pA/pF, *cha^B19^>ChR2* vs *cha^B19^>ChR2; VAChT^12Q^*, respectively, *p* = 0.81). Notably, expression of wild-type VAChT also increased optogenetically-evoked EPSC duration (485.4 ± 33.0 vs 636.4 ± 44.6 ms, *cha^B19^>ChR2* vs *cha^B19^>ChR2; VAChT*, respectively, *p* = 0.02; Fig. 2*C*). Again, with no effect on amplitude (26.4 ± 2.5 vs 29.0 ± 2.4 pA/pF, *p* = 0.49).

We also measured the amplitude ratio between the first and second EPSC evoked at a following frequency of 1 Hz. The resulting ratios (EPSC2/EPSC1) were 75.2 ± 6.9% vs 93.0 ± 1.2%, *cha^B19^>ChR2* versus *cha^B19^>ChR2; VAChT^12Q^*, respectively, *p* = 7 × 10^−3^ (Fig. 2*A*,*B*). Whereas overexpression of wild-type *VAChT* did not statistically differ from control (75.2 ± 6.9% vs 87.3 ± 2.6%, *p* = 0.13) This effect was abrogated when stimulation frequency was reduced to once every 20 s (0.05 Hz; 84.8 ± 4.2% vs 89.1 ± 3.0%, *cha^B19^>ChR2* vs *cha^B19^>ChR2; VAChT^12Q^*, respectively, *p* = 0.92). We rationalize that this reduction represents an inability to fully refill recycled SVs and thus represents a net reduction in quantal content of the second SRC. That this reduction is greatest in wild type (Fig. 2*A*) is in agreement with our observations above; that expression of *VAChT^12Q^* increases the fill load of SVs. This effect is mitigated using a lower frequency of stimulation (0.05 Hz), which we predict provides sufficient time to fully recycle/re-fill SVs. Taken together, and in line with previous literature (Cash et al., 2016), our data suggest that expression of *VAChT* ^12Q^ is sufficient to increase loading of SV in the terminals of cholinergic central neurons, an effect that was not observed through upregulation of the wild-type (13Q) transporter (Cash et al., 2016). This is sufficient to produce mEPSCs exhibiting larger amplitudes, SRCs/EPSCs exhibiting longer durations and the ability of the presynaptic terminals to resist synaptic depression following continuous 1-Hz evoked vesicle release.

### VAChT^14Q^ decreases SV loading at cholinergic synapses

To investigate the contribution to SV loading made by the VAChT polyQ region, we created two CRISPR knock-in gene replacements. The first extended the polyQ region by one additional glutamine (VAChT^14Q^), while the second deleted the polyQ region (VAChT^ΔQ^). A third CRISPR was attempted containing a single glutamine truncation (VAChT^12Q^) to validate our findings using the GAL4/UAS system described above. However, despite several injection attempts (BestGene and Manchester Fly Facility) we were unable to generate transgenic progeny. CRISPR mutations were confirmed not to increase *VAChT* transcript expression relative to wild type. qRT-PCR determination of expression level (using relative fold change: Log_2_) was: VAChT^ΔQ^ (0.63 ± 0.24, *n* = 2, *p* = 0.89) and VAChT^14Q^ (0.82 ± 0.88, *n* = 2, *p* = 0.83) compared to control lines (set to 0, *n* = 3).

**Figure 3.**
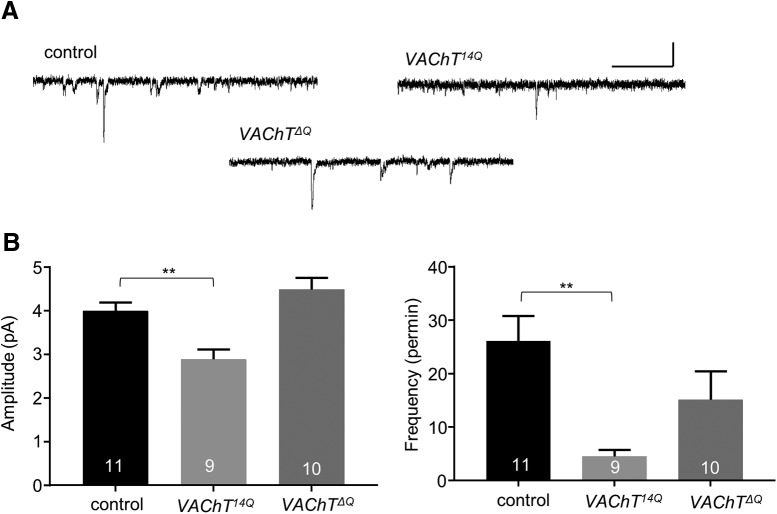
*VAChT* polyQ manipulation alters spontaneous neurotransmission. ***A***, Representative traces of mEPSCs recorded from embryonic late stage 17 aCC/RP2 between control, *VAChT^14Q^*, and *VAChT^ΔQ^.* Scale bar: 3 pA/30 ms. ***B***, VAChT^14Q^ mutants display significantly reduced mEPSC amplitude (*p* = 0.005) and frequency (*p* = 0.005). However, no obvious difference in mEPSC kinetics is observed in VAChT*^ΔQ^*mutants for either amplitude (*p* = 0.37) or frequency (*p* = 0.24). All data points are mean ± SEM, *n* stated in each bar.

**Figure 4.**
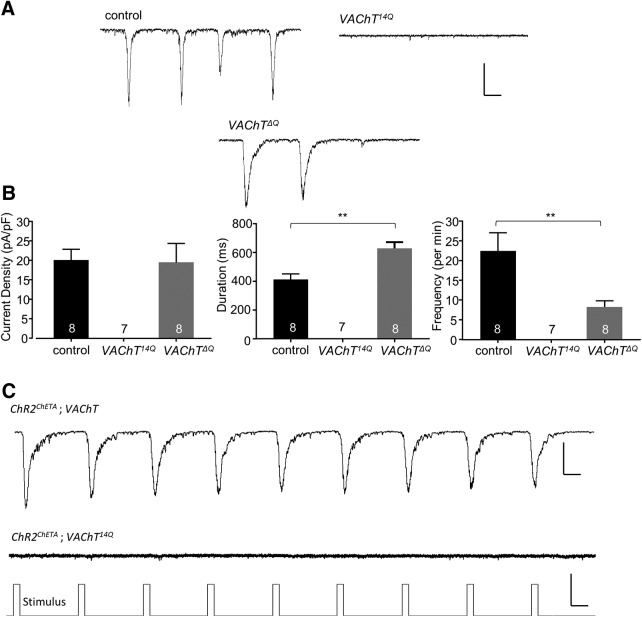
*VAChT* polyQ manipulation alters evoked neurotransmission. ***A***, Representative traces of SRCs recorded from aCC/RP2 between control, VAChT^14Q^, and *VAChT^ΔQ^*. Scale bar: 50 pA/300 ms. Data points are mean ± SEM, *n* stated in each bar. ***B***, VAChT^14Q^ mutants lack any observable SRCs. By contrast, VAChT*^ΔQ^* mutants show SRCs with no observable change in amplitude (*p* = 0.91). However, VAChT*^ΔQ^* mutants exhibit increased SRC duration (*p* = 0.003) and reduced SRC frequency (**p = 0.01). Control: ***C***, Representative traces (from a total of four experiments) of ChR2^ChETA^ evoked EPSCs recorded from RP2 between control (upper trace) and *VAChT^14Q^* (lower trace). Scale: 50 pA/300 ms (upper), 2 V/300 ms (lower).

Homozygous VAChT^14Q^ is embryonic lethal. Embryos develop normally until late stage 17, identified by the presence of inflated trachea, clearly visible mouth hooks, normal gross CNS morphology and body-wall musculature. However, no coordinated peristaltic waves of body-wall muscles were observed indicative of a failure of the central motor network. Recordings from aCC/RP2, in late stage 17 embryos, showed that mEPSC amplitude was significantly reduced (4.0 ± 0.2 vs 2.9 ± 0.2 pA, control vs *VAChT* ^14Q^, respectively, *p* = 5 × 10^−3^) as was frequency (26.1 ± 4.8 vs 4.56 ± 1.1 per min, control vs *VAChT* ^14Q^, respectively, *p* = 5 × 10^−3^; Fig. 3*A*,*B*). This effect was opposite to that observed following expression of *VAChT* ^12Q^. Note: the absolute amplitude and frequency shown in Figure 3 differs from the equivalents shown in Figure 1 (*VAChT* ^12Q^) because the developmental stage differs (embryo vs L3). Remarkably, knock-in of *VAChT^14Q^* does not support evoked SRCs (Fig. 4*A*,*B*). Combining ChR^ChETA^ in this background was also unable to evoke optogenetically-evoked EPSCs (Fig. 4*C*). We rationalize that the reduction in mEPSC amplitude observed, indicative of insufficient loading of cholinergic SVs, is sufficient to prevent evoked release.

Homozygous knock-in of *VAChT^ΔQ^* produces viable larvae. However larval development ceases during L1 after which lethality occurs. Recordings from late stage 17 embryonic aCC/RP2 motoneurons, in homozygous *VAChT^ΔQ^*, shows no obvious effects to either mEPSC amplitude (4.0 ± 0.2 vs 4.5 ± 0.3 pA, control vs *VAChT^ΔQ^*, respectively, *p* = 0.37) or frequency (26.1 ± 4.8 vs 15.1 ± 5.3 per min, control vs *VAChT^ΔQ^*, respectively, *p* = 0.24; Fig. 3*A*,*B*). Unexpectedly, we did observe a change to endogenous SRC kinetics. Specifically, SRC duration was increased (411.7 ± 38.4 vs 627.9 ± 44.5 ms, control vs *VAChT^ΔQ^*, respectively, *p* = 3 × 10^−3^), and frequency reduced (22.4 ± 4.7 vs 8.3 ± 1.6 per min, control vs *VAChT^ΔQ^*, respectively, *p* = 0.01). SRC amplitude was not affected (20.2 ± 2.8 vs 19.6 ± 4.9 pA/pF, control vs *VAChT^ΔQ^*, respectively, *p* = 0.91; Fig. 4*A*,*B*). The lack of effect to mEPSC amplitude suggests that the number of glutamines in the polyQ region is a more important determinant, rather than the presence or absence of this region.

## Discussion

We report neurophysiological consequences arising from the manipulation of the C-terminal *VAChT* polyQ region. We find, in agreement with previously published literature, that the presence of *VAChT^12Q^* (i.e., truncating the polyQ region by one glutamine) increases both amplitude and frequency of mEPSCs at identified central cholinergic synapses. This increase in ACh loading may explain the increased duration in evoked SRCs also observed. Conversely, we further show that a CRISPR- induced single amino acid extension of this region (*13Q* to *14Q*) results in reduced amplitude and frequency of mEPSCs and an associated inability to support evoked release. Finally, CRISPR mediated deletion of the polyQ region (13Q to 0Q) does not affect mEPSC kinetics showing elongation or truncation of the polyQ region is more detrimental to cholinergic release than removal of this region. This work highlights the *VAChT* polyQ region as an important determinant mediating cholinergic loading in *Drosophila*.

It is notable that although mEPSC amplitude is increased following expression of *VAChT^12Q^* the effect to SRCs is limited to increased duration. We speculate that this may be indicative that the postsynaptic nAChR receptor field is already fully saturated under endogenous conditions and heightened cholinergic tone, through *VAChT^12Q^* upregulation, is thus restricted to increasing SRC duration. Similarly, we can only speculate on why increased SRC duration is accompanied by a decrease in SRC frequency. A possible explanation is a homeostatic-type negative feedback mechanism which acts to dampen the activity of presynaptic interneurons that form the central pattern generator controlling locomotor output. Future experiments will be required to clarify these issues.

Our results suggest that the length of the polyQ domain is both deterministic for SV filling and for probability of SV release. Reducing glutamines by one residue is sufficient to increase SV load and release probability and vice versa. Moreover, addition of a glutamine (14Q) is sufficient to remove the ability of the CNS to generate a rhythmic fictive locomotor pattern, which is reliant on evoked release. We rationalize that *VAChT^14Q^* disrupts cholinergic loading, generating partially-filled SVs that, in turn, prevent evoked synaptic release. By contrast, increasing SV loading (12Q) results in evoked release events of longer duration. These observations are in agreement with recent work using a light activated vATPase pump (pHoenix) localized to SVs (Rost et al., 2015). Rost and colleagues used this tool to show that glutamatergic vesicles are only ‘nearly full’ under normal conditions (i.e., can be further filled) and, moreover, show vesicle load is proportional to release probability (Rost et al., 2015). Our data are supportive of this observation: only increased SV loading supports evoked release. Moreover, our results are also indicative of a set point model, in which vesicles can only release once they surpass a threshold load. This hypothesis, proposed by Williams in 1997, proposed two distinct models of SV loading. The set-point model proposes a mechanism restricting the amount of neurotransmitter per vesicle to a fixed maximum, whereas, the steady state model suggests the amount of neurotransmitter that enters a SV is offset by leakage, but that both are independent variables that can autonomously change to produce SVs with variable levels of filling (Williams, 1997). The set point model is consistent with observations at the snake NMJ and *Drosophila* central neurons (Parsons et al., 1999; Cash et al., 2016). Whereas, the steady state model better describes loading at murine and *Drosophila* NMJ and in mammalian cell culture models (Varoqui and Erickson, 1996; Song et al., 1997; Daniels et al., 2004; Wilson et al., 2005; Prado et al., 2006; De Castro et al., 2009; Lima et al., 2010).

Analysis of related *Drosophila* spp. reveal polyQ regions of differing lengths (e.g., nine in *D. willastomi*, 11 in *D. simulans*, and 15 in *D. pseudoobscura*). It is tempting to speculate that evolution may have manipulated the length of the polyQ region to alter SV content in these related species. However, recordings from aCC/RP2 in these related species show mEPSC amplitude is remarkably conserved (S. W. Vernon and R. A. Baines, unpublished data). Thus, the predicted effect of SV loading due to change in polyQ length, across these related species, may have been abrogated by compensatory mutations in other regions of the VAChT. A comparative analysis may thus be useful to identify such regions for future study.

The VAChT polyQ region is specific to insects. A BLAST search comparison shows no other insect neuronal vesicular transporter possesses a C-terminal polyQ domain (S. W. Vernon and R. A. Baines, unpublished data). Mammalian VAChT possesses a di-leucine motif in the same approximate location to the insect polyQ domain. The di-leucine motif is well established as a trafficking region (Bonifacino and Traub, 2003). Removal of the mammalian VAChT C-terminal tail, or specific mutation of the di-leucine motif, results in mislocalization of the transporter to the neuronal membrane (Colgan et al., 2007). Mutant Htt protein containing a polyQ expansion from 20Q to 120Q was found to preferentially bind to SVs in murine axon terminals and, further, to displace the binding of Huntington-associated protein (HAP1) usually co-localized to SVs (Li et al., 2003). 120Q mutants were also shown to reduce glutamate release suggesting a direct interaction between extended polyQ domains and synaptic release (Li et al., 2003). HAP1 has also been shown to bind synapsin 1 (Mackenzie et al., 2016) which is critical for SV pool mobilization and formation (Rosahl et al., 1995; Akbergenova and Bykhovskaia, 2010). We therefore theorize that the polyQ region in VAChT may play a similar role in trafficking the transporter to the SV, plasma membrane and/or SV pool formation.

It is notable that complete removal of the *VAChT* polyQ region does not influence mEPSCs, although does alter SRC kinetics (increasing their duration). This dichotomy may mirror an increasingly accepted molecular distinction between spontaneous (mEPSCs) and synchronous (SRC/EPSC) release modalities (Sara et al., 2005; Ramirez and Kavalali, 2011; Kavalali, 2015). Other work has shown, for example, that mEPSC release is maintained in the absence of the vesicle associated SNARE protein synaptobrevin, while evoked release is halted (Schoch et al., 2001). Munc-13 has also be shown to influence the spatial localization of evoked release while having no effect on mEPSCs at *C. elegans* NMJ (Zhou et al., 2013). These observations are predictive of a model in which multiple fusion complexes are physiologically separate and dependent on the modality of release. Moreover, a role for *VAChT* in SV release is indicated by a reported interaction between synaptobrevin and *VAChT.* A glycine to arginine substitution (*G342R*) in *VAChT* is sufficient to reduce cholinergic mediated larval motility in *C. elegans*, an effect that is rescued by a complimentary substitution of an isoleucine to an aspartate in synaptobrevin (Sandoval et al., 2006).

*VAChT^ΔQ^* mutants show early larval mortality (L1) despite being able to produce SRCs. This is further confused by the similarity in SRC kinetics with *cha^B19^*>VAChT^12Q^ which produce viable L3 larvae and adults. We attribute early *VAChT^ΔQ^* mortality to the lack of wild-type transporter present in the *VAChT^ΔQ^* genetic background and may be consistent with cholinergic deficiencies presented in wider physiologic function. In humans, ChAT immunoreactivity and nAChR/mAChR expression is observed in non-neuronal epithelial, endothelial, mesothelial and immune cells (Wessler and Kirkpatrick, 2008) and are shown to modulate multiple cellular processes including but not exclusive to, cellular migration and apoptosis (Grando et al., 2006), proliferation (Metzen et al., 2003), anti/proinflammatory responses (Shytle et al., 2004; Pavlov and Tracey, 2005) and histamine release (Reinheimer et al., 2000; Wessler and Kirkpatrick, 2008). In insects, non-neuronal ACh has been shown to be heavily influential in reproduction and larval development (Wessler et al., 2016; Wessler and Kirkpatrick, 2017) and so it remains possible that VAChT modulation may alter wider, and currently unknown, physiologic aspects of larval development.

The effects we report here relating to expression of *VAChT^12Q^* (truncation) versus *VAChT^14Q^* (expansion) were achieved using different experimental conditions. *VAChT^12Q^* was tested using Gal4-based overexpression in an otherwise wild-type *VAChT* background, while *VAChT^14Q^* was tested using a CRISPR mutant. This was because our attempt to make *VAChT^12Q^* via CRISPR was unsuccessful. Thus, the results we report here must be tempered. Indeed, the co-presence of wild-type *VAChT* in *VAChT^12Q^* upregulation may, to some extent, reduce the observed phenotype. Moreover, protein level, nor protein localization, was measured and thus the possibility remains that the VAChT^14Q^ mutation may affect expression levels and/or vesicular localization, which makes it difficult to reach firm conclusions about results obtained. However, we do not believe this detracts from the interpretation of the data presented within this study.

Since the first demonstration of fixed quanta that describes spontaneous release of SVs, a key question of “how does a SV know when it is full” remains to be answered. The polyQ region of the *Drosophila VAChT*, that we report here, seemingly orchestrates the filling of cholinergic SVs at central synapses. Future studies to identify the function of this region, including identification of binding partners, provide optimism for understanding how SVs monitor their fill state.

## Synthesis

Reviewing Editor: Douglas Bayliss, University of Virginia School of Medicine Decisions are customarily a result of the Reviewing Editor and the peer reviewers coming together and discussing their recommendations until a consensus is reached. When revisions are invited, a fact-based synthesis statement explaining their decision and outlining what is needed to prepare a revision will be listed below. The following reviewer(s) agreed to reveal their identity: NONE. Note: If this manuscript was transferred from JNeurosci and a decision was made to accept the manuscript without peer review, a brief statement to this effect will instead be what is listed below.

The reviewers find that this paper contains interesting results and that the authors have responded well to earlier comments. However, whereas the authors provide new qRT-PCR data suggesting similar overall expression levels of the 14-Q mutant, there is no direct evidence that the protein is correctly localized to the vesicles. Thus, the revised paper should explicitly acknowledge the possibility that the 14-Q mutation may affect expression levels and/or vesicular localization of the VAChT, which makes it difficult to reach firm conclusions about those results.
